# Survival benefits of hypofractionated radiotherapy combined with temozolomide or temozolomide plus bevacizumab in elderly patients with glioblastoma aged ≥ 75 years

**DOI:** 10.1186/s13014-019-1389-7

**Published:** 2019-11-12

**Authors:** Makoto Ohno, Yasuji Miyakita, Masamichi Takahashi, Hiroshi Igaki, Yuko Matsushita, Koichi Ichimura, Yoshitaka Narita

**Affiliations:** 1 Department of Neurosurgery and Neuro-Oncology, National Cancer Center Hospital, 5-1-1, Tsukiji, Chuo-ku, Tokyo, 104-0045 Japan; 2 Department of Radiation Oncology, National Cancer Center Hospital, 5-1-1, Tsukiji, Chuo-ku, Tokyo, 104-0045 Japan; 3 Division of Brain Tumor Translational Research, National Cancer Center Research Institute, 5-1-1, Tsukiji, Chuo-ku, Tokyo, 104-0045 Japan

**Keywords:** Elderly glioblastoma, Hypofractionated radiotherapy, Temozolomide, Bevacizumab, Vulnerability

## Abstract

**Background and purpose:**

The purpose of this study was to evaluate the outcomes of elderly patients (aged ≥75 years) with newly diagnosed glioblastoma (GBM), who were treated with hypofractionated radiotherapy comprising 45 Gy in 15 fractions combined with temozolomide (TMZ) or TMZ and bevacizumab (TMZ/Bev).

**Materials and methods:**

Between October 2007 and August 2018, 30 patients with GBM aged ≥75 years were treated with hypofractionated radiotherapy consisting of 45 Gy in 15 fractions. Twenty patients received TMZ and 10 received TMZ/Bev as upfront chemotherapy. O-6-methylguanine DNA methyltransferase *(MGMT)* promoter methylation status was analyzed by pyrosequencing. The cutoff value of the mean level of methylation at the 16 CpG sites was 16%.

**Results:**

Median overall survival (OS) and progression-free survival (PFS) were 12.9 months and 9.9 months, respectively. The 1-year OS and PFS rates were 64.7 and 34.7%, respectively. Median OS and PFS did not differ significantly between patients with *MGMT* promoter hypermethylation (*N* = 11) and those with hypomethylation (*N* = 16) (17.4 vs. 11.8 months, *p* = 0.32; and 13.1 vs. 7.3 months, *p* = 0.11, respectively). The median OS and PFS were not significantly different between TMZ (*N* = 20) and TMZ/Bev (*N* = 10) chemotherapy (median OS: TMZ 12.9 months vs. TMZ/Bev 14.6 months, *p* = 0.93, median PFS: TMZ 8.5 months vs TMZ/Bev 10.0 months, *p* = 0.64, respectively). The median time until Karnofsky performance status (KPS) score decreasing below 60 points was 7.9 months. The best radiological responses included 11 patients with a partial response (36.7%). Grade 3/4 toxicities included leukopenia in 15 patients (50%), anorexia in 4 (13.3%), and hyponatremia during concomitant chemotherapy in 3 (10%).

**Conclusion:**

Our hypofractionated radiotherapy regimen combined with TMZ or TMZ/Bev showed benefits in terms of OS, PFS, and KPS maintenance with acceptable toxicities in elderly patients with GBM aged ≥75 years.

## Introduction

Glioblastoma (GBM) is the most aggressive type of primary brain tumor. It predominantly affects elderly patients; the median age at diagnosis is 67 years, and 16.3% of patients diagnosed with GBM in Japan are older than 75 years [1]. Age is one of the most consistently reported prognostic factors and survival is shown to decline with increasing age [2, 3]. The 1-year relative survival rates in the United States are 29.3 and 12.2% for patients with GBM aged 65–74, and those aged ≥75 years, respectively [4]. Thus, the outcome of older patients is extremely poor.

In the management of elderly patients with GBM, hypofractionated radiotherapy has been investigated due to its advantage of having a shorter treatment time [5]. A trial comparing treatment with 40 Gy in 15 fractions to standard radiotherapy consisting of 60 Gy in 30 fractions did not show any differences in overall survival (OS), suggesting that the short-course radiation therapy (RT) was not an inferior treatment option [6]. The Nordic trial, which randomly assigned patients with newly diagnosed GBM who were aged 60 years and older to receive TMZ monotherapy, hypofractionated radiotherapy of 34 Gy in 10 fractions, or standard radiotherapy of 60 Gy in 30 fractions, demonstrated that hypofractionated radiotherapy produced survival rates that were similar to those in patients receiving standard radiotherapy [7].

In 2017, Perry et al. reported a phase III study showing that the addition of TMZ to short-course radiotherapy (40 Gy in 15 fractions) resulted in longer survival than radiotherapy alone in patients aged 65 years or older, thereby establishing TMZ combined with radiotherapy at 40 Gy in 15 fractions as the standard therapy in elderly patients with GBM [8]. However, in this study the median age was 73 years, and only 30% of patients were aged > 75 years; therefore, the survival impact of this chemoradiation therapy regimen on older patients (> 75 years) remains unclear [9]. Moreover, there have been no comparative studies comparing the regimen of 40 Gy in 15 fractions or other hypofractionated regimens in combination with TMZ, therefore, optimal dose-fractionation schedules in combination with TMZ remains unclear [10]. Furthermore, the advantage of addition of bevacizumab (Bev) to TMZ combined with hypofractionated radiotherapy has not been fully evaluated [11–13]. Therefore, optimal treatment remains unclear especially in patients aged ≥75 years.

We hypothesized that TMZ combined with dose-intensified RT with the same fractionation as the regimen of 40 Gy in 15 fractions may improve patient outcomes. In addition, because elderly patients are known to have more angiogenic tumors than those who are younger [14], upfront addition of Bev to TMZ may have an advantage. We determined a dosage of 45 Gy in 15 fractions as our exploratory regimen based on a previous report that used a 15-fraction schedule [15] combined with TMZ, or TMZ plus Bev (TMZ/Bev).

In this study, we retrospectively analyzed the treatment outcomes of patients with newly diagnosed GBM aged 75 years of age or older, treated with hypofractionated radiotherapy consisting of 45 Gy in 15 fractions, combined with TMZ or TMZ/Bev to evaluate the feasibility of this treatment among a cohort of older patients (aged ≥75 years).

## Patients and methods

### Patient characteristics

Forty-two patients aged ≥75 years were diagnosed with GBM at our center between October 2007 and August 2018. Among these patients, 12 were excluded from this study for various reasons (RT alone in 5 patients, RT consisting of 60 Gy in 30 fractions in 4, TMZ alone in 1, RT and Bev, and refusal of therapy in 1). The remaining 30 patients who underwent initial surgery followed by hypofractionated radiotherapy consisting of 45 Gy in 15 fractions combined with TMZ or TMZ/Bev were included in the study. All patients were diagnosed with GBM by neuropathologists at our hospital according to the revised 4th edition of the World Health Organization classification scheme [16].

The patient’s clinical, operative, and radiological information were reviewed, and the following data were collected for each: clinical history, date of initial operation, postoperative adjuvant therapy regimen, use of corticosteroids at the start of RT, date of tumor recurrence, date of recording a Karnofsky performance status (KPS) score of 50, date of death or last hospital visit, extent of resection, number of TMZ cycles, reason for TMZ discontinuation, and treatment after tumor recurrence. The extent of resection was determined based on the surgeons’ operative notes and on postoperative imaging studies, classified as either total if 100% of the enhanced lesion was resected, subtotal if 95–99% was resected, partial if < 94% was resected, or as a biopsy.

### Treatment

After GBM diagnosis, all patients received radiotherapy with concomitant and adjuvant TMZ or TMZ/Bev. Since June 2013, Bev has been approved as a treatment option for GBM, and thereafter upfront TMZ/Bev was administered to patients with KPS scores of 70 or less, as well as patients with KPS scores of 80 who had residual tumors.

The TMZ dose was 75 mg/m^2^/day during radiotherapy and 150–200 mg/m^2^/day for 5 days every 28 days when administered as adjuvant treatment for a maximum of 24 cycles (until December 2013) or 12 cycles (since January 2014), or until disease progression. The dose of Bev was 10 mg/kg every 2 weeks or 15 mg/kg every 3–4 weeks. Blood testing was performed every week in the concomitant phase, and every month in the adjuvant phase.

All patients received hypofractionated radiotherapy consisting of 45 Gy at the isocenter in 3 Gy daily fractions using a 3-dimensional conformal radiotherapy technique with 4 or more portals, including non-coplanar beams in general. Target volumes were determined based on postoperative magnetic resonance imaging (MRI) fused into the treatment planning computed tomography (CT) images; preoperative MRI was also utilized for correlation analysis. The gross tumor volume (GTV) was defined as the residual tumor on contrast-enhanced T1-weighted images. The clinical target volume 1 (CTV1) was defined as the GTV, the surgical cavity, and hyper-intensity area on T2-weighted images or fluid attenuated inversion recovery (FLAIR) images with a 1.5 cm margin. The CTV2 was defined as the GTV plus the surgical cavity with a 1.5 cm margin. The planning target volumes 1 and 2 were defined as the CTV1 and CTV2 plus a 0.5 cm margin, respectively. PTV1 was irradiated up to 30 Gy in 10 fractions, and boost irradiations of 15 Gy to the PTV2 were followed. Irradiation fields were modified, if necessary, through dose-volume histogram evaluation for the organs-at-risk such as the brainstem, optic nerve and chiasm, retina, and lens. The maximum dose to the brainstem, retina, optic nerve and chiasm were restricted to 40 Gy. The lens was shielded by multileaf collimators when possible through the beam’s eye view.

### Response evaluation

Patients were evaluated using T2-weighted or FLAIR imaging and contrast-enhanced T1-weighted imaging within 3 days before and 1 day after surgery; follow-up imaging via MRI was performed at the time of the completion of RT, 1 month thereafter, and every 2 months thereafter or according to clinical symptom development. When pseudoprogression was suspected and the lesions were stable, the patients were observed without changing adjuvant chemotherapy; patents with lesions that resolved during follow-up were considered to have pseudoprogression.

Disease progression was evaluated according to the Response Assessment Criteria for High-Grade Gliomas (RANO criteria) [17]. Briefly, complete response (CR) was defined as the complete disappearance of all enhancing measurable and non-measurable lesions for at least 4 weeks. Partial response (PR) was defined as a ≥ 50% decrease in lesion size compared to baseline, as measured by summing the products of the perpendicular diameters of all measurable lesions that were sustained for at least 4 weeks, with no new lesions or any progression of non-measurable disease observed. Progressive disease (PD) was defined as a ≥ 25% increase in the sum of the products of the perpendicular diameters of the enhancing lesions, compared with the smallest tumor measurement, a significant T2-weighted or FLAIR non-enhancing lesion, the appearance of any new lesions, definite clinical deterioration not attributable to causes other than the tumor, or death. If there was uncertainty regarding whether there was progression, close observation or C11-methionine positron emission tomography (Met-PET) was performed. If follow-up imaging studies showed progressive enlargement, tumor recurrence was diagnosed. If low-uptake on Met-PET was observed, then tumor necrosis was diagnosed [18]. Stable disease (SD) was defined has not meeting any of the criteria for CR, PR, or PD. Patients who underwent total resection and did not have measurable lesions were also classified as having SD [17].

### IDH1/2 mutation analysis

Nucleic acids were extracted from tumors using the DNeasy Blood & Tissue Kit (Qiagen, Maryland, USA), according to the manufacturer’s instructions. The mutation hotspots on codon 132 of *IDH1* and codon 172 of *IDH2* were assessed through pyrosequencing, as previously described [19, 20]. The pyrosequencing assays were designed to detect all known mutations on these codons [19, 20]*.*

### Immunohistochemistry

Immunohistochemistry using a mouse monoclonal anti-human isocitrate dehydrogenase 1 (IDH1) R132H antibody (Dianova, Hamburg, Germany) (clone: H09, dilution 1:50) was performed using the EnVision FLEX system (Dako Japan Inc., Tokyo, Japan) (polymeric method), an automatic staining machine [21].

### MGMT promoter methylation analysis

The methylation status of the *MGMT* promoter was analyzed using bisulfite modification of the tumor genomic DNA followed by pyrosequencing, as previously described [20]. Methylation was considered positive when its mean level at the examined 16 CpG sites was greater than 16% [20].

### Statistical analysis

OS was defined as the interval between the date of surgery and that of death. Progression-free survival (PFS) was defined as the interval between the date of surgery and that of the detection of progression. The time until KPS score decrease to below 60 was defined as the interval between the date of surgery to that of the first recording of a KPS score of 50. The patients’ OS, PFS, and time until the KPS score dropped below 60 were calculated using the Kaplan-Meier method and compared using the log-rank test. Analyses were conducted using JMP® 8 (SAS Institute Inc., Cary, NC, USA) and GraphPad Prism® version 6.0 (GraphPad Software, La Jolla, California, USA). In all tests, probability values of < 0.05 were considered statistically significant.

## Results

### Patient characteristics and treatments

The patient characteristics are summarized in Table 1. The median age was 80 years (range: 75–87 years), and the median KPS score was 70. Twelve patients (40.0%) underwent biopsy, and the reasons to perform biopsy included multifocal tumors in 3 patients, deep seated tumors in 5, and eloquent localization in 4 patients. Twenty patients (66.7%) received TMZ and 10 (33.3%) received TMZ/Bev as upfront chemotherapy. The median number of TMZ cycles was 5 (range: 0–20 cycles). Most patients (21 of the 28 patients [75.0%]) who experienced tumor recurrence terminated treatment and received supportive care only. Twenty-nine tumors were classified as *IDH1/2* wildtype by pyrosequencing (*N* = 27) or immunohistochemistry (*N* = 2). Eleven tumors (36.7%) were classified as having hypermethylation and 16 (53.3%) as having hypomethylation (Table 1).

**Table 1:** Patient characteristics

	No. (%) of Patients
Age (yr)	
Median	80
Range	75–87
Sex	
Male	19 (63.3)
Female	11 (36.7)
Karnofsky performance status	
100, 90	2 (6.7)
80, 70	17 (56.7)
60, 50	11 (36.7)
Extent of resection	
Total resection	8 (26.7)
Subtotal resection	3 (10)
Partial resection	7 (23.3)
Biopsy	12 (40)
Chemotherapy	
Temozolomide	20 (66.7)
Temozolomide/Bevacizumab	10 (33.3)
Adjuvant Temozolomide cycles (n)	
Median	5
Range	0–20
Use of corticosteroid	
Yes	6 (20)
No	24 (80)
Treatment after tumor recurrence	
Best supportive care	21 (70)
Bev	5 (16.7)
Surgery followed by Temozolomide	1 (3.3)
Temozolomide	1 (3.3)
No recurrence	2 (6.7)
MGMT promoter status	
High	11 (36.7)
Low	16 (53.3)
N/A	3 (10)
IDH 1/2 status	
Wild-type	29 (96.7)
Mutant	0
N/A	1 (3.3)

*IDH* Isocitrate dehydrogenase, *MGMT* O6-methylguanine DNA methyltransferase, N/A Not available

### Outcomes

**Fig. 1:**
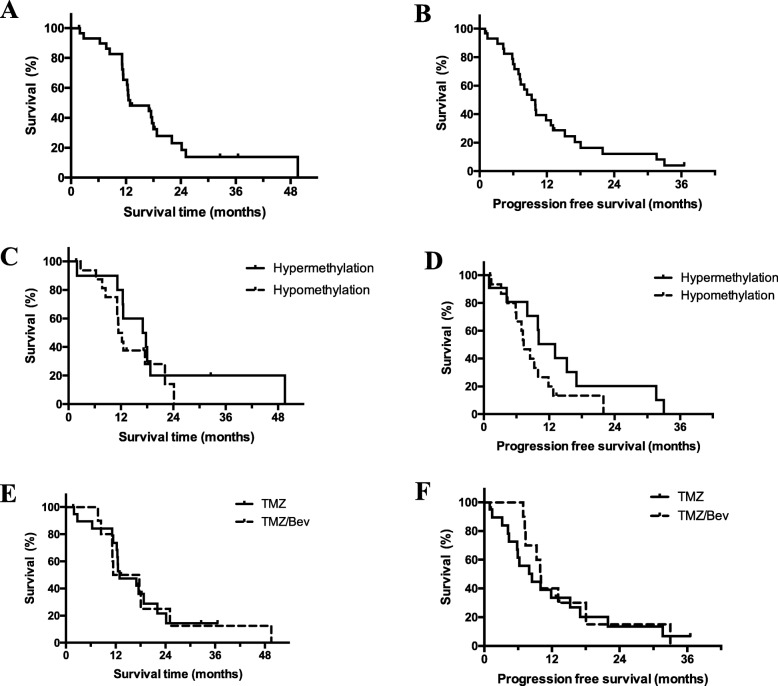
Kaplan-Meier curve of overall survival (**a**, **c**, **e**), progression-free survival (**b**, **d**, **f**). **a** The median survival time was 12.9 months. **b** The median progression-free survival was 9.9 months. **c** The median survival times were 17.4 months in patients with *MGMT* promoter hypermethylation (*N* = 11), and 11.8 months in those with *MGMT* promoter hypomethylation (*N* = 16) (*p* = 0.32). **d** The median progression-free survival was 13.1 months in patients with *MGMT* promoter hypermethylation (*N* = 11), and 7.3 months in those with *MGMT* promoter hypomethylation (*N* = 16) (*p* = 0.11). **e** The median survival times were 14.6 months in patients treated with temozolomide plus bevacizumab (*N* = 10), and 12.9 months in those treated with temozolomide (*N* = 20) (*p* = 0.93). **f** Median progression-free survival was 10.0 months in patients treated with temozolomide plus bevacizumab (*N* = 10), and 8.5 months in those treated with temozolomide (*N* = 20) (*p* = 0.64). Abbreviations: *MGMT* O-6-methylguanine DNA methyltransferase, *TMZ* temozolomide, *Bev* bevacizumab

**Fig. 2:**
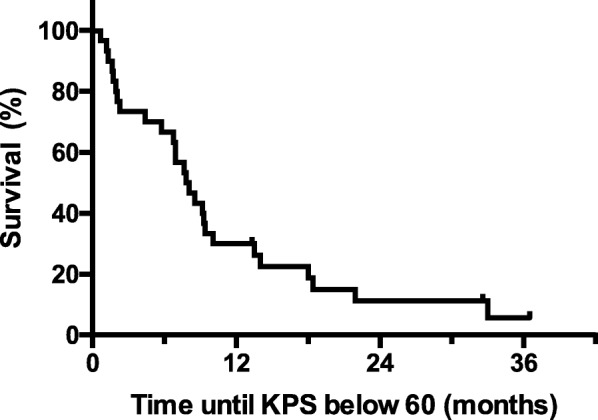
Kaplan-Meier curve of time until the decrease in Karnofsky performance status score (KPS) to below 60. The median time until KPS score decreased to below 60 was 7.9 months. Abbreviations: *KPS* Karnofsky performance status

**Fig. 3:**
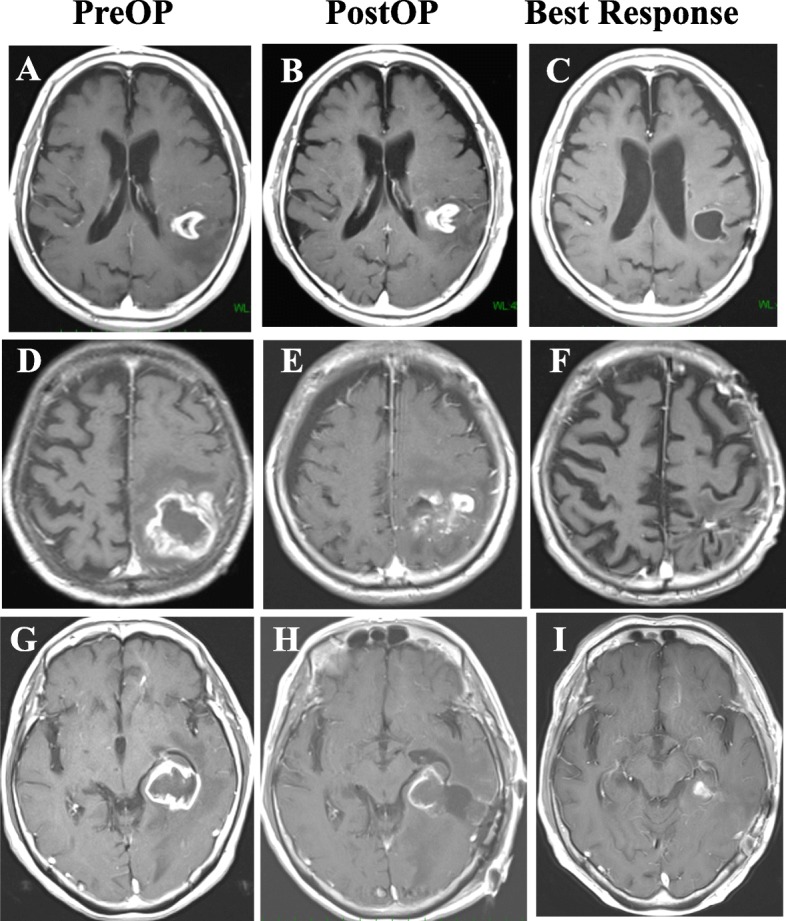
Three representative patients whose T1-weighted magnetic resonance images with gadolinium enhancement showed a partial response. **a** An 80-year-old man presented with a left temporo-parietal enhanced tumor and underwent biopsy. **b** Afterwards, he received temozolomide combined with radiotherapy consisting of 45 Gy in 15 fractions and showed a remarkable response with cystic degeneration. **c** The tumor was *MGMT* promoter-hypomethylated. **d** A 78-year-old man presented with a left parietal enhanced tumor and underwent partial resection. **e** Afterwards, he received temozolomide combined with radiotherapy consisting of 45 Gy in 15 fractions and nearly achieved a complete response. **f** The tumor was *MGMT* promoter-hypermethylated. **g** A 77-year-old woman presented with a left temporal enhanced tumor and underwent partial resection. **h** Afterwards, she received temozolomide combined with radiotherapy consisting of 45 Gy in 15 fractions and achieved tumor size reduction. **i** The tumor was *MGMT* promoter-hypomethylated. Abbreviations: *MGMT* O-6-methylguanine DNA methyltransferase

The median OS was 12.9 months and the median PFS was 9.9 months (Fig. 1a and b, respectively). The 1-year OS and PFS rates were 64.7 and 34.7%, respectively. Notably, 5 patients lived for more than 2 years after operation. The median OS in patients with *MGMT* promoter hypermethylation (*N* = 11) was 17.4 months, whereas the median OS in patients with *MGMT* promoter hypomethylation (*N* = 16) was 11.8 months; the difference was not statistically significant (*p* = 0.32) (Fig. 1c). The median PFS in patients with *MGMT* promoter hypermethylation and hypomethylation were 13.1 and 7.3 months, respectively; again, the difference was not statistically significant (*p* = 0.11) (Fig. 1d). The median OS and PFS were not significantly different between TMZ (*N* = 20) and TMZ/Bev (*N* = 10) chemotherapy (median OS for TMZ vs. TMZ/Bev: 12.9 months vs. 14.6 months, respectively; *p* = 0.93; median PFS for TMZ vs TMZ/Bev: 8.5 months vs. 10.0 months, respectively; *p* = 0.64) (Fig. 1e and f, respectively). The median time until KPS score decrease to below 60 was 7.9 months (Fig. 2). The best radiological responses were PR in 11 patients (36.7%), SD in 12 (40%), and PD in 7 (23.3%); none achieved CR. Pseudoprogression was observed in 1 patient. There was one case, which showed slowly progressive paraventricular disease with low-uptake on Met-PET. We considered this to be a case of radiation necrosis. Three representative patients with PR are described in Fig. 3.

All patients discontinued TMZ or TMZ/Bev maintenance therapy because of tumor progression (*N* = 10; 33.3%), clinical deterioration without radiological tumor progression (*N* = 9, 30.0%), completion of 12 cycles in (*N* = 3; 10.0%), complications (*N* = 4;13.3%), and switching to palliative care at a different hospital (*N* = 4; 13.3%) (Table 2).

**Table 2:** Reasons of temozolomide or temozolomide plus bevacizumab maintenance therapy discontinuation

	*N* = 30
Tumor progression	10 (33.3%)
Clinical deterioration without radiological tumor progression	9 (30.0%)
Completeion of 12 cycles	3 (10.0%)
Complication	4 (13.3%)
Changing hospital for palliative care	4 (13.3%)

### Toxicities

Toxicities are summarized in Table 3. In the concomitant phase: 15 patients (50%) experienced grade 3/4 leukopenia, 4 (13.3%) had neutropenia, 4 (13.3%) had anorexia, 3 (10.0%) had hyponatremia, and 4 (13.3%) exhibited skin rashes. Among the 24 patients who received adjuvant chemotherapy, 11 (45.8%) experienced grade 3/4 leukopenia, 1 (4.2%) had neutropenia, 1 (4.2%) had anemia, and 2 (8.3%) had skin rashes. One patient discontinued TMZ/Bev chemotherapy owing to gastrointestinal hemorrhage. One other patient experienced severe ulceration of the skin of his left leg during TMZ/Bev chemotherapy and terminated treatment.

**Table 3:** Summary of Grade 3 or 4 toxicities

Category	Concomitant (*N* = 30)	Adjuvant (*N* = 24)
Hematologic		
Neutropenia	4 (13.3%)	1 (4.2%)
Lymphocytopenia	15 (50%)	11 (45.8%)
Thrombocytopenia	1 (3.3%)	0
Anemia	1 (3.3%)	1 (4.2%)
Hepatic		
Aspartate transaminase	2 (6.7%)	0
Alanine transaminase	2 (6.7%)	1 (4.2%)
Pulmonary (pneumonitis)	2 (6.7%)	1 (4.2%)
Anorexia	4 (13.3%)	1 (4.2%)
Hyponatremia	3 (10.0%)	0
Hypokalemia	1 (3.3%)	0
Skin rash	4 (13.3%)	1 (4.2%)

## Discussion

The treatment of elderly patients with GBM is challenging. Although TMZ combined with hypofractionated RT of 40 Gy in 15 fractions is established as the standard treatment in elderly patients aged ≥65 years, optimal treatment remains unclear, especially in patients aged ≥75 years. The European guidelines state that patients with GBM with *MGMT*-methylation aged ≥75 years may receive hypofractionated RT/TMZ or TMZ alone, and those with GBM without *MGMT*-methylation may receive hypofractionated RT alone, indicating that either RT or TMZ alone might be a treatment option [22]. As the elderly population expands, the incidence of GBM in these elderly patients will increase, and the development of effective and safe treatment to improve outcomes in this patient population becomes increasingly important.

A few studies investigated the treatment outcomes in older patients (aged ≥75 years) with GBM. In a phase III trial, Perry et al. showed the survival benefit of adding TMZ to short-course radiotherapy in patients with GBM (aged ≥65 years) and demonstrated the survival benefit of TMZ among patients aged ≥76 years (median OS 10.0 months [RT/TMZ] vs. 7.6 months [RT alone]) [8]. Uzuka et al. analyzed 79 patients with GBM (aged ≥76 years) and reported that the median OS and PFS were 9.8 months and 6.8 months, respectively. Multivariate analysis demonstrated post-operative KPS and TMZ therapy to be significant predictors of survival [23]. Harris et al. retrospectively examined the outcome of 108 patients with GBM (aged ≥75 years) and reported a median OS of 13.3 months, 6.3 months, and 1.9 months in patients who underwent RT/TMZ, RT alone, and best supportive care, respectively. They demonstrated that chemotherapy was the only positive prognostic factor on multivariate analysis and concluded that all patients should be considered for TMZ [24]. Our results that the median OS and PFS were 12.9 and 9.9 months, respectively, and that the 1-year OS and PFS rates were 64.7 and 34.7%, respectively, are comparable with these studies and support the benefit of chemoradiation therapy in these elderly patients.

Various regimens using hypofractionated radiotherapy combined with TMZ for the treatment of elderly patients with GBM have been investigated. Such regimens include 30 Gy in 6 fractions, 40 Gy in 15 fractions, 45 Gy in 15 fractions, and 35 Gy in 10 fractions. The resulting OS and PFS range intervals were 5.4–20 months, and 3.5–9.6 months, respectively (Table 4) [8, 13, 15, 24, 25, 27–29, 31, 33]. The biologic radiation response is predicted by using the linear-quadratic model (LQ model); generally, the tumor is considered an acutely responding tissue with high α/β ratios, whereas normal tissue (including normal brain) is late-responding with low α/β ratios [34]. According to the LQ model, our regimen (45 Gy in 15 fractions) is characterized as having a greater dose-intensity than the regimen of 40 Gy in 15 fractions, with less impact than the regimen of 60 Gy in 30 fractions. Terasaki et al. treated 26 patients with TMZ combined with hypofractionated radiotherapy of 45 Gy in 15 fractions and reported median OS and PFS of 15.6 and 9.6 months, respectively, with a response rate of 34.6%. In considering the above together with our result, the regimen of 45 Gy in 15 fractions seems to be favorable, although it would not be superior to the standard regimen of 60 Gy in 30 fractions [28]. Roa et al. reported no significant survival differences between 40 Gy in 15 fractions and 25 Gy in 5 fractions in the elderly or frail patients with GBM, suggesting that the α/β ratio of GBM could be lower than 2–3 Gy [30]. The low α/β ratio of GBM supports the advantage of the hypofractionated approach and further studies are needed to develop the optimal dose-fractionation schedule, which meets efficacy and safety.

**Table 4:** Summary of currently published studies using hypofractionated radiotherapy and temozolomide for elderly patients with glioblastomas

Author	Treatment	n	Patients	OS (mo)	PFS (mo)
Minniti et al. 2009 [33]	RT 30Gy/6fr + TMZ	43	Age ≥ 70 and KPS ≥60	9.3	6.3
Uto et al. 2015 [31]	RT 35Gy/10fr + TMZ	11	Age ≥ 70	13.2	7
Minniti et al. 2012 [29]	RT40Gy/15fr + TMZ	71	Age ≥ 70 and KPS ≥60	12.4	6
Perry et al. 2017 [8]	RT40Gy/15fr	281	Age ≥ 65 and PS 0–2	7.6	3.9
	RT40Gy/15fr + TMZ	281		9.3	5.3
Lombardi et al. 2015 [28]	RT40Gy/15fr + TMZ	71	Age ≥ 65 and PS 0–2	13.8	N/A
	RT60Gy/30fr + TMZ	166		19.4	
Chang-Halpenny et al. 2015 [25]	RT 35Gy/10fr + TMZ	29	Age ≥ 65	5.4	N/A
	RT60Gy/30fr + TMZ	100		13	
Terasaki et al. 2011 [15]	RT45Gy/15fr + TMZ	26	median 61(39–79)	15.6	9.6
Lim et al. 2015 [27]	RT45Gy/15fr + TMZ	33	Age ≥ 70Age < 70 and PS ≥ 3 or biopsy or rapid growth	10.6	7.5
Harris et al. 2017 [24]	Best supportive care	31	Age ≥ 75	1.9	N/A
	RT alone*	38		6.2	N/A
	RT* + TMZ	33		13.2	N/A
Matsuda et al. 2018 [13]	RT45Gy/15fr + TMZ, Bev after recurrence	18	Age ≥ 75	20	2.5
Present study	RT45Gy/15fr + TMZ or TMZ/Bev	30	Age ≥ 75	12.9	9.9

*RT* Radiation therapy, *TMZ* Temozolomide, *Bev* Bevacizumab, *PFS* Progression free survival, *OS* Overall survival, *KPS* Karnofsky performance status, *PS* Performance status, N/A not available

* Radiation therapy was either hypofractionation (40Gy), or longer-course (60Gy)

Bevacizumab, an antiangiogenic monoclonal antibody, which binds to vascular endothelial growth factor (VEGF), received approval in 2013 as a treatment for both primary and recurrent GBM in Japan. The potential benefits of Bev in elderly patients with GBM have been suggested [11–13]. Babu et al. demonstrated that elderly patients with GBM experienced significantly longer survival after treatment with Bev than those without Bev (20.1 months vs. 7.9 months: *p* < 0.0001). They also suggested that Bev could be an independent favorable prognostic factor after adjusting for age, KPS, and extent of resection [11]. Matsuda et al. showed that Bev prolonged overall survival time after tumor recurrence and suggested the usefulness of Bev in a salvage setting in elderly patients with GBM [13]. Although Bev was selectively used in our study for patients with lower performance status or residual tumors, their OS and PFS were comparable to those of patients with higher performance status or extensive tumor removal. This observation suggests the potential benefit of upfront Bev in the elderly patients.

*MGMT* promoter methylation status has been reported to be a prognostic factor and a predictor of TMZ treatment efficacy in elderly patients [7, 32]. However, in our study, we found no statistically significant differences in either OS or PFS according to *MGMT* promoter methylation status, although PFS tended to be longer in patients with *MGMT*-methylation than in those without. Similarly, Biau et al. reported that *MGMT* promoter methylation was neither a prognostic nor a predictive factor in a real-life, unselected elderly patient population [35]. In our study, the small number of patients and intratumoral heterogeneity [36] may explain the lack of statistical significance. Our results suggest the potential benefit of using TMZ, irrespective of *MGMT* promoter methylation status.

In our cohort, we had 5 patients who lived for more than 2 years. The possible reasons of these favorable outcomes might be multifactorial, including total removal, high performance status, upfront Bev, and *MGMT* hyper methylation. Our cases suggest that there might be some patients who have great benefit from the aggressive treatment in spite of their advanced age.

The quality of life or in-house independence is an important treatment goal for elderly patients with GBM. Chinot et al. reported that the median time of maintaining a KPS score of 70 or higher in adult patients with GBM was 9.0 months in their radiotherapy/TMZ/Bev-treated group and 6.0 months in their radiotherapy/TMZ-treated group [37]. In comparison, the median time for maintaining a KPS score of 60 or higher in our study was 7.9 months, which seems to be favorable in taking into consideration their elderly patient population. Our results suggest that our treatment approach could enable elderly patients to maintain their activities of daily living for longer periods.

Nine patients terminated TMZ or TMZ/Bev maintenance treatment owing to clinical deterioration without radiological evidence of tumor progression, as did 4 owing to complications. Clinical deterioration and the development of complications are difficult to predict before treatment; moreover, most patients in our study (75%) terminated treatment after their first tumor recurrence and received only supportive care. These observations exemplify the vulnerability of elderly patients. To minimalize the treatment risk, such vulnerabilities must be investigated, and appropriate selection criteria must be devised to better identify patients with treatment-related vulnerabilities.

Treatment-related toxicities are a main concern for elderly patients treated for GBM. We observed grade 3/4 leukopenia in half of our patients; 10–13% of our patients also experienced anorexia, hyponatremia, and skin rash during TMZ or TMZ/Bev treatment. All these toxicities were manageable, and anorexia was related to the TMZ; hyponatremia and hypokalemia were related to the anorexia. Skin rashes in 4 patients were assumed to be drug-related (i.e due to an anticonvulsant), and one skin rash was caused by Herpes Zoster virus infection. Thrombocytopenia was observed in only one patient, which is lower than that of the other study by Perry, et al. [8]. Two of our patients also experienced severe non-hematological toxicities (gastrointestinal hemorrhage, and skin ulceration, respectively), which we assumed to be a consequence of Bev administration. As such, physicians should remain cognizant of the potential risk of these uncommon but severe complications, and thus observe their patients (especially the elderly among them) more carefully.

Our study had certain limitations. First, this work was retrospective, and we did not provide data regarding the quality of life during the course of treatment. Second, our cohort was too small to draw definitive conclusions; however, most of our patients were followed from the time of their surgeries to their terminal stages at our institute, thereby providing robust clinical data.

In conclusion, our hypofractionated radiotherapy and upfront Bev combined with TMZ approach appears to be feasible with favorable outcomes and acceptable toxicities in patients aged ≥75 years. Further studies are needed to develop the optimal dose-fractionation schedule and combination with chemotherapy in the elderly patients with GBM.

## Data Availability

The datasets used and/or analyzed during the current study are available from the corresponding author on reasonable request.

## Abbreviations

Bev: Bevacizumab
CR: Complete response
CTV: Clinical target volume
FLAIR: Fluid-attenuated inversion recovery
GBM: Glioblastoma
GTV: Gross tumor volume
IDH: Isocitrate dehydrogenase
KPS: Karnofsky performance status
MGMT: O-6-methylguanine DNA methyltransferase
MMSE: Mini-Mental State Examination score
MRI: Magnetic resonance imaging
OS: Overall survival
PD: Progressive disease
PFS: Progression-free survival
PR: Partial response
RANO: Response Assessment Criteria for High-Grade Gliomas
SD: Stable disease
TMZ: Temozolomide
